# Hospital and Institutional News

**Published:** 1912-04-13

**Authors:** 


### HOSPITAL AND INSTITUTIONAL NEWS.

### THE INSURANCE ADVISORY COMMITTEE.

Glancing at the names of the members of the advisory committee appointed under the National Insurance Act, to advise and assist the joint committee with the making and alteration of regulations under Part I. of the Act, we see that of the 159 members thirty-three are medical men. Of these thirteen have been nominated by the British Medical Association, three by the Association of Registered Medical Women, while seventeen have been selected by the Commissioners. Among other persons selected by the Commissioners are two hospital authorities, namely, Dr. D. J. Mackintosh, M.B., M.V.O., of the British Hospitals Association, and Mr. William West, of the Central Hospital Council for London. Dr. Mackintosh is, of course, medical superintendent of the Western Infirmary, Glasgow, and Mr. West is a treasurer of St. George's Hospital and a hospital architect. We publish on page 48 the names of the medical members, who are divided into two groups, "qualified medical practitioners with personal experience of general practice" and "medical men selected by the Commissioners."

### THE NEW SUPERINTENDENT OF FRIMLEY SANATORIUM.

Dr. Marcus Paterson's successor as Medical Superintendent at Frimley Sanatorium on his appointment as director of the anti-phthisis campaign in Wales is Dr. Wilfrid Ombler Meek. Dr. Meek, who is a St. Thomas's man, where he has held the post of assistant director of the clinical laboratory, graduated M.B., B.S. at London University, and has devoted much time and study to pulmonary tuberculosis. His publications include "Tuberculous Endocarditis."

### LORD LISTER'S WILL AND BEQUESTS.

The estate of Lord Lister has been valued for probate at £65,190 net. His bequests for public purposes include £10,000 to King Edward's Hospital Fund, to King's College Hospital, to the North London and University College Hospital, and to the Royal Society. He added with characteristic modesty that he did not wish his name to be "in any way associated with these sums in the future." He left also £20,000 to the Lister Institute for Preventive Medicine, and requested his nephews, Mr. Rickman John Godlee and Mr. Arthur Hugh Lister, to arrange his scientific papers and sketches, with power to destroy or to dispose of such as they deem of no scientific value. As the result of their arrangements, the residue deemed of permanent importance has been left to the Royal College of Surgeons of England and his orders and medals to the University of Edinburgh, which is instructed to dispose of all or any of them, "for example by having the medals melted down or the diplomas or other writings destroyed" should they desire at any time to do so. It will strike the most casual observer that for a man who was regarded by Europe as the head of his profession, who received almost every decoration and distinction which his country and scientific bodies throughout the world had to offer, £65,000 is not an immense fortune to have acquired. Similar success in the world of commerce would have made its protagonist a multimillionaire. The prizes of a learned profession like medicine are rarely financial, and such monetary wealth as even the most successful practitioner acquires is never given to the scientist. Nevertheless, those who knew Lister and his tastes will never quarrel with this result. His aims were not commercial, and his reward cannot be measured by an auditor's account of financial profit and loss.

### THE KING'S FUND AND LORD ROSEBERY.

The honorary secretaries of King Edward's Hospital Fund have sent us a reply which we print in full elsewhere to the complaint which, as noted in these columns, Lord Rosebery saw fit to reiterate at the annual meeting of the Royal National Hospital for Consumption, Ventnor. After remarking that the primary object of the Fund is to help institutions which are in need, the honorary secretaries proceed: "The accounts furnished by the Ventnor Hospital in support of its three recent applications to the Fund showed excesses of income over expenditure of £3,331 in 1907, £7,152 in 1909, and £958 in 1910. It is true that these surpluses were due to legacies, and that in the case of a hospital which receives exceptional legacies in a given year, or which has so little free capital that the investment of any unspent legacies is a matter of financial urgency, the existence of such a surplus would not be decisive against a grant being made. But in the case of this hospital the average surplus for the six years (1905 to 1910) for which the figures have been submitted to the Fund was £2,119 on an average annual expenditure of £13,356, while in the year last mentioned the free capital amounted to £63,428, besides endowments of £8,945. In only one of these years did the figures show a deficit, and in the year in which those accounts were published the hospital did not apply to the Fund." The "deaf ears" to which Lord Rosebery appealed conclude by adding: "All this Lord Rosebery could have ascertained for himself if his other numerous engagements had permitted him to attend any of the meetings of the King's Fund, of which he has been a member since 1908."

### IMPORTANT NEW INFIRMARY AT BRISTOL.

A very important step has been taken by the Bristol Guardians in provisionally accepting the tenders for the proposed conversion of Southmead Workhouse into an infirmary for the sick poor of Bristol. As Mr. J. T. Francombe, Vice-Chairman of the Guardians, pointed out in an admirable summary of the history of the scheme, last June a scheme was submitted for utilising the three existing workhouses in order to improve the classification and the accommodation at relatively small cost. The new ward blocks to be added to Southmead

Workhouse will give some 400 beds, or a total accommodation for the sick of about 500. The kitchen and the laundry are also to be improved, and this will have the effect of classifying the imbeciles and the chronic aged sick at Stapleton, while Eastville will be used purely as a workhouse for the able-bodied. In the infirmary there will be 144 new beds for men, 250 for women, 20 maternity and 18 children's beds, or, including the old wards, 210 for men, 252 for women, 28 maternity and 54 children's beds, making a grand total of 544. The cost will be under £50,000. Mr. W. S. Skinner is the architect. Thus a very important Poor Law hospital will be added to Bristol's institutions, which is also administratively interesting, as it has been decided finally that instead of the large double pavilions of three storeys first suggested, one double pavilion and three single pavilions each of two storeys have been adopted for their convenience in working. This new step towards classification and towards the complete separation of the infirmary from the workhouse is very important, and it may be expected that Bristol's example in the matter will influence other Guardians to adopt the same course, which is the progressive and finally inevitable one.

### DR. WALDO ON POST-MORTEM FEES.

The recent appeal of a surgeon of a London hospital after having given evidence at an inquest in the City led Dr. Waldo, the Coroner, once more to point out the anomaly that medical men are paid adequate fees in every court except the coroners' courts. As Dr. Waldo has made many prominent efforts to secure the alteration of the law, notable among which was his evidence before the Departmental Committee, we hope that his advice to the medical man, to ask the two members for the City, Mr. Arthur Balfour and Sir Frederick Banbury, to take the matter up in Parliament, will be followed. Luckily the total neglect of the medical profession by Parliament and politicians is a degree less absolute than it was before the Insurance Act was introduced. Now or never, therefore, is the time for the profession to exploit in every possible way the hearing they have begun to exact in the House and from its members.

### AN ODD GIFT TO THE ROYAL FREE HOSPITAL.

The motives which lead chance subscribers to allocate their gifts to hospitals are tacitly recognised as incalculable by the growing practice of advertising institutions in the same general way as business concerns, and how unexpected may be the influence which leads to a subscription has been seen lately. Mr. Reginald Garratt, secretary of the Royal Free Hospital, has announced the receipt of two guineas as a protest against Sir Almroth Wright's recent reference to medical women in the lay newspapers. The donor, one of the criticised sex, stated that many other women who have reason to bless women doctors would probably wish to follow her example. This confession, however, is enough to explain the lady's motive in picking out, as the motor omnibuses remind us, the only hospital in London with a medical school for women. At all events, that women doctors do secure a large following is a fact which Sir Almroth Wright and other critics cannot ignore, and the power of satisfying patients is in the long run the test of all medical skill.

### PRESENTATION AT NORTHAMPTON.

Dr. F. Buszard, who has now completed fifty years of service at the Northampton General Hospital, to which he was appointed house surgeon in 1862, has been presented with his portrait. The artist is Mr. Harris Brown, who has painted a further portrait for the hospital, and the presentation was made by Lord Northampton. Dr. Buszard in his formal reply took occasion to urge the importance of maintaining the voluntary system. If the possibility of the hospitals passing under public control be realised, he thought the best that could be hoped for was that the present condition of things should become stereotyped. This is only another way of saying that the voluntary spirit would be lost inevitably.

### AN ABANDONED PROJECT AT GRAVESEND HOSPITAL.

Among the improvements badly needed at the Gravesend Hospital are the redecoration of the wards in the north block and the modernisation of the laundry. These improvements have now been postponed for want of money, and as their completion is felt by the committee to be of serious importance, and has been admitted for a year or two as due or overdue, the decision to postpone them is a depressing, if necessary, one. Such expenditure as ward-redecoration, whether placed to the establishment or to the extraordinary-expenditure account, is not a very popular need to plead for. Nevertheless, the part that room-decoration should be made to play in the treatment of the sick was laid down in forcible but delightfully pointed terms by Florence Nightingale, who was never tired of insisting on the value of cheerful colours and harmonious surroundings to the sick. No detail in this respect was held by her to be too small to receive attention. She used to point out how valuable was so apparently trivial a matter as the occasional change of a picture on the wall to those in bed. In her hands establishment charges of this sort became human kindnesses as obvious as the smoothing of a pillow or the brisk quietness of a tread. We hope, therefore, that the supporters of this hospital in or around Gravesend will be induced to realise this, and that Mr. Walter Pearson, the secretary, will receive enough support to proceed at once with the redecoration.

### NEW MEMBER OF THE MIDWIVES BOARD.

Professor Briggs has been appointed a member of the Central Midwives Board for seven years from the expiration of the term of service of Sir William Sinclair. He is Professor of Midwifery and Gynæcology in the University of Liverpool, surgeon to a hospital, and hon. medical officer to the Maternity Hospital, Liverpool. The honour done the city through him is appreciated warmly.

### A CAREER IN THE PUBLIC HEALTH SERVICE.

The Medical Officer of Health for St. Albans, Dr. John Morison, M.D., has died at Thirlestane, his house. His student days were passed at Guy's and University College Hospitals, but he graduated at Edinburgh in 1865. Ten years later he gained the diploma of Public Health at Cambridge. His hospital appointments at Jersey General Hospital were succeeded by his public health work at St. Albans, which is interesting to the hospital world from the amount of extra-official work which he was able to combine with it. For instance, he was medical superintendent to the Sisters' Hospital for Infectious Diseases, a consulting physician to the St. Albans and Mid-Herts Hospital, and a medical officer to several schools, besides being a public vaccinator. Apart from this Dr. Morison published "Inorganic Evolution" and contributed to the Transactions of the Hertfordshire Natural History Society, of which he was formerly president. The public health service, apart from its intrinsic advantages, has surely many inducements in the light of such a many-sided career as Dr. Morison's.

### ALLEGED COTTAGE HOSPITAL SCANDAL.

At a specially called meeting of the present and recent subscribers to the Woolwich and Plumstead Cottage Hospital, presided over by the Mayor, Mr. S. H. Cuff, a resolution was moved by Mr. George Whale: "That in order to restore public confidence in the management of the Woolwich and Plumstead Cottage Hospital, it is desirable that the present members of the committee and the honorary secretary should resign office, and that they be requested so to do in May next, and to call a special meeting then of the subscribers in order to elect a new committee and honorary secretary." The resolution was carried with one dissentient. In supporting it, Mr. Whale said it was significant that the members of the house committee had signed the notice convening the meeting. "All the facts were not known, but enough were known from the committee's minutes and from certain correspondence to render such drastic measures as those he had moved the only course. The hospital was 'nominally managed' by fifteen members and four trustees, but it could scarcely be said to be managed by the committee at all."

### THE CHARGES AGAINST THE SECRETARY.

It seemed to him that "the affairs of the hospital had been left far too largely to the honorary secretary, Mr. Woodford, who was well aware that this meeting was taking place but was not present. The honorary secretary had by notice of motion without reasons alleged attempted to rescind the appointment of the treasurer to the committee." Mr. Whale proceeded: "The secretary 'made violent personal attacks upon the chairman' and went 'hawking the office of chairman round the committee,' and all the time there was a chairman in office in the person of the Rev. C. A. Berry. The secretary overrode the committee's decisions and decided who should receive notices to attend the meetings and who should not. The rules of the institution, framed many years ago, were ambiguous and inadequate to the present position. Though two or three of the committee distinguished themselves by opposing a certain purchase of land, the majority would have to share the responsibility of the secretary's conduct, and a few of them even attempted to justify that conduct. The minutes showed a series of petty personal quarrels and hardly referred to finance from beginning to end, not even to who drew the cheques and authorised payments. King Edward's Hospital Fund had now ceased to make any subscription to the Woolwich Hospital. The cost per bed was £88 12s. 11d. There were many other instances of the gross mismanagement of the finances. A site it was proposed to purchase was found eventually to be owned by the honorary secretary himself, and it appeared from the minutes, which were not quite clear, that a valuer was appointed on behalf of the honorary secretary, and he said the site was worth £2,150. Supposing it was the best site, which he doubted, the proper course was for the secretary to stand aside as the vendor of the land. On inquiry it had been decided not to buy this site, but to extend the present buildings." He added that the chairman was entitled to their thanks for the resolute stand he had made.

### THE DEFENCE AND THE CHARITY COMMISSIONERS.

The matter was about to be put to the vote when Mr. Kimber intervened. He said that none of Mr. Whale's charges against the committee was of the slightest value. The matter was before the Charity Commissioners, and the Charity Commissioners was the proper tribunal. The whole agitation had been got up by Colonel Leslie, who had surreptitiously taken and was detaining the minute-book. Alderman Syer supported Mr. Kimber. The chairman of the Hospital Saturday Fund, Mr. Kemp, said that nine months' experience on the committee enabled him to support everything Mr. Whale had said. After further discussion the resolution, as stated already, was carried, and also a further one which appointed a Town Hall committee to take the necessary steps to secure more efficient management, and called on the committee, trustees, secretary, and treasurer to afford this committee all facilities for ascertaining the facts. It was also decided to send copies of the resolutions to the honorary secretary, Mr. Woodford, and to the Charity Commissioners. In view of the latter's pending inquiry comment would serve no useful purpose, but it is at least satisfactory that so far no want of care in respect of the patients has been alleged.

### A DISEASE PECULIAR TO MINERS.

Within the last four or five years various industrial diseases, of whose very existence the bulk of the community was previously ignorant, have become familiar topics. An excellent example is miner's nystagmus, in which the late Mr. Simeon Snell did so much to arouse public interest and professional research. It appears, however, that colliery medical officers have long been familiar with this complaint, and that unobtrusively its prevention has been laboriously investigated. From a correspondence just published between the Home Office and Sir A. B. Markham, it is apparent that the former is alive to the magnitude and wide distribution of this disease, and is zealous in following such clues as its scientific advisers can provide, for finding a means of prevention. Dr. Court, of Staveley, is a physician in a colliery district, and his forty years' experience has convinced him that the true causal factor is not, as most authorities have supposed, the constrained positions in which the miner at the coal face has to work, but rather the feeble illumination provided by the usual patterns of safety-lamp. Sir A. B. Markham forwarded an account of Dr. Court's hypothesis to the Home Office, and it is interesting to note that it has since been completely confirmed by Drs. Llewellyn and Haldane, who have been inquiring into the disease on behalf of that Department. It is to be hoped that these important discoveries will speedily be tested by the adoption of improved patterns of lamp, and that the result will be a steady decrease in the incidence of this disabling and expensive industrial disease.

### THE BRITISH MEDICAL ASSOCIATION'S PROGRAMME.

The item most interesting to the hospital world in the programme for the eighteenth annual meeting of the British Medical Association in Liverpool is that contained in the medical sociological section, when "A public medical service under professional control" and "Payment of medical services by capitation *versus* payment per attendance under the National Insurance Act" will be the subjects discussed. It is perhaps regrettable that the representative meeting of the Association will not begin till July 19th, and the annual general meeting will therefore take place on July 23rd, when the President, Sir James Barr, will deliver his address in the evening. By the time the meetings take place the Insurance Act will presumably be in force in some form or other, in precisely what still depends upon the vigour with which the medical profession through its Association, the hospitals through their Association, and the great Funds continue to impress their views upon the Government. It is most important that no section of the hospital world should allow even such momentous public occurrences as the coal strike to exclude public attention from their attitude, and the more the general course of events tends to obliterate the impression which has been already made, the more must the hospital world press home its demands if the Government is not to use public interest in other matters as a screen behind which to shelter themselves from the demands of the profession and the hospitals.

### A NEW HOSPITAL FOR CAMDEN TOWN.

The new out-patient department of the Hampstead General and North-West London Hospital, designed by the architects Messrs. Young and Hall, was opened by Princess Christian of Schleswig-Holstein last week. The new unit consists of a waiting-hall for 200 patients, consulting, ophthalmic and $x$-ray rooms, a casualty-room, an isolation ward, a dispensary, and a small mortuary. The cost, including equipment, has been £9,000, towards which the King's Fund has given or promised more than half; and the staff will consist of two resident medical officers and three nurses. This new building has a special interest because it was designed to take the place of the North-West London Hospital in Kentish Town, the institution which, of course, was amalgamated a few years ago with the Hampstead General Hospital on Haverstock Hill. The out-patient department in Kentish Town will now be closed, and while the new unit, which stands in Baynham Street, Camden Town, is intended principally for out-patients, emergency cases demanding operative treatment will be met by the Hampstead Hospital staff, which will be sent for by telephone. Mr. C. Walton Sawbridge, acting-chairman of the council, improved the occasion last week by a brief historical retrospect. This new unit demands attention as being a foreshadowing of these casualty and sorting centres, which eventually will replace the existing out-patient departments, and will allow all ward cases to be treated in buildings removed from the noise and bad air of town streets.

### THE PRITCHARDS AND BRISTOL EYE DISPENSARY.

At the end of the present year the Bristol Eye Dispensary will have completed a century of existence, and its president (Mr. J. P. Worsley), who has been connected with it ever since it became a public institution, revived some interesting memories last week. Many, if not most, of these are associated with the name of Pritchard. The founder, Mr. J. B. Estlin, an oculist himself, started the dispensary in 1812 by the informal but exacting process of giving up certain hours in the mornings for the poor who came to consult him. His great-nephew, Mr. Pritchard, is now a member of the medical staff, while another great-nephew, Mr. E. A. Pritchard, for several years has acted as honorary secretary to the dispensary. The success of the institution may be judged from the fact that the number of patients increased to a total of 3,000 last year, partly due to the numbers of school children sent by the Education Committee, which, Mr. A. W. Pritchard remarked, should appoint a paid oculist. If this were not done the dispensary might have to shut its doors against the school children.

### SURGEONS' COSTUMES IN THE THEATRE.

On retiring from the position of senior honorary surgeon to the Royal Infirmary, Liverpool, Mr. F. T. Paul has been presented with his portrait painted in oils by Mr. G. Hall Neale. Mr. Paul has been associated with the Infirmary for thirty-eight years. Dr. R. C. Brown, who was one of the speakers at the ceremony, in alluding to the costumes which present-day surgeons often affected in the operating-theatre, is reported to have prophesied that the day was coming when they would be seen in tights, with no creases for microbes to enter and lurk in. Mr. Paul, who began life in a London office, was formerly a student at Guy's.

### THE ST. BARTHOLOMEW'S HOSPITAL APPEAL.

Mr. Edwin S. Layton, a member of the Court of the Stationers' Company and a Governor of St. Bartholomew's Hospital, is acting as honorary secretary of the Special Appeal Committee, which has still a heavy task in front of it. Up to the present £3,000 have been promised in annual subscriptions, and £6,000 have been received as donations. As we pointed out early in February when the appeal was issued, some £10,000 increased income is required, and a sum of £100,000 to liquidate its liabilities and to free it from bank overdrafts and interest on loans. It is to be hoped every effort will be made to raise the necessary money, and, as we pointed out previously, "provided every care be taken to avoid any recurrence of the causes which have contributed to the present *impasse*," we have no doubt that the collective action of the City of London and of the Governors of St. Bartholomew's Hospital will relieve the famous City hospital of its very serious financial difficulties.

### THE TORQUAY ISOLATION HOSPITAL ON TRIAL.

Some months ago we referred to the charges against the management of the Torquay Isolation Hospital which were brought to light in the action taken by ex-Sergeant Gregory against the Corporation in consequence of the death of his son at the hospital, when neglect by the staff was alleged. A jury at the County Court awarded £50 damages, against which, as we subsequently stated, an appeal was lodged, when the Corporation pleaded successfully the Public Authorities Protection Act. A demand was also made for costs, but Judge Lush-Wilson deprived the Corporation of these in two of the hearings. The litigation has been so much prolonged that the case has been virtually *sub judice* for months, and therefore no comment could be rightly or usefully made in our columns. We have never lost sight of the case however, which is of wide institutional importance, and it is satisfactory to hear that a Local Government Board inquiry is virtually certain. It is now stated that this inquiry will refer to other details of the management of this Isolation Hospital, the charges against which are too serious to be left in a state of uncertainty any longer.

### AT A SCOTCH INFECTIOUS HOSPITAL.

Mr. Wellwood Maxwell has been re-elected chairman of the committee of management of the Stewartry Infectious Diseases Hospital, Dumfries. During the year there has been maintained a large increase in the number of patients treated at the hospital, and the days in hospital for 1911 reached the high figure of 3,505. In these circumstances the charge for maintenance against the local authorities has been kept at 1s. 6d. per day. It is noteworthy that a large section of the patients were scarlet-fever cases. The causes of the maintained increase in the total number of patients were not mentioned at the annual meeting, but it must always be remembered that notification has been much more rigidly followed of late, and a numerically recorded increase—such, for instance, as cancer appears to give—is a tribute rather to the modern statistics than to the incidence of the disease.

### OUT-PATIENTS AND SOLICITORS' TOUTS.

"Truth" draws attention to the case of a correspondent who wrote to that paper stating that after an accident he was taken to St. Mary's Hospital. A day or two later the writer apparently was written to by a certain T. Pearce, from an address in the Gray's Inn Road, exactly opposite to the Royal Free Hospital, tenanted by a "Legal Aid Society." *Truth* has previously commented upon the means by which these legal aid societies have obtained the names and addresses of patients from St. Mary's, and now adds: "Really the hospitals ought to see that their accident wards are not made the happy hunting ground for solicitors' touts." This indictment adds another if not a new terror to out-patient administration, and may make conscientious almoners alarmed at the further investigation demanded of them. But what if these solicitors' touts come in the guise of out-patients? It is to be presumed that the active control of well-organised out-patient departments precludes the presence of individuals whose business is not recognisable as that of the sick. What experience or knowledge of the above-mentioned gentlemen have our out-patient clerks and medical officers?

### HULL AND HOSPITAL SUNDAY.

The Mayor of Hull, Mr. T. S. Taylor, has been able to announce that his appeal to make up the Hospital Sunday collections to £1,500 has been successful, and further, that an additional £500 contingent on this result has been received from Mr. G. F. Grant in accordance with his promise. The honorary secretaries of the fund, Canon Lambert and the Rev. R. Batey, have been much thanked for their strenuous work, and Hospital Sunday in Hull, if somewhat late in the day, has been dragged from the slough into which it was falling. That a measure of success has been retrieved by the Mayor's special effort must be very satisfactory to him and to the hospitals, but in view of next year's Hospital Sunday a question still presents itself. Is it really desirable that Hospital Sunday should owe its success to other inspiration than that of the pulpits and the chaplains? Is it certain that every succeeding Mayor, even if he have the same sympathy with Hospital Sunday as Mr. Taylor has, will have the same opportunity for assisting it? In short, should not the clergy of Hull realise that a Mayor's appeal to second their own efforts must not be counted on as an annual resource? Hospital Sunday primarily is the special privilege of the clergy, the supreme occasion on which they plead the cause of the good Samaritan. This year they have found in Mr. Taylor a good Samaritan themselves, and their gratitude that so good a cause as theirs on Hospital Sunday has been in the end successful will be best expressed next year by ensuring that its success shall be their glory alone.

### NEW WING AT CARDIFF HOSPITAL.

Part of the new wing of King Edward VII. Hospital, Cardiff, was opened to receive patients for the first time last week. It comprises the wards associated with the names of Mr. W. James Thomas and Mrs. John Nixon, and the St. David's Ward, given by the Marquis and Marchioness of Bute, and the accommodation has been increased by twenty-five beds. It is much to be hoped that the remaining fifty beds in the new wing will be available shortly, and that the apparent end of the coal strike will produce a new batch of subscriptions as a thankoffering.

### THE OUT-PATIENT DEPARTMENT AT ST. THOMAS'.

Coinciding with the fact that for the first time since the present building was erected all the wards have been in use at St. Thomas', Mr. S. S. Wainwright, the treasurer, feels that a new out-patient department is the need of most importance to-day. There were not far short of 90,000 new out-patients last year, and though such figures cannot be understood, except in relation to the accommodation available, we may take Mr. Wainwright's word for it that unless the Insurance Act should materially reduce the numbers of out-patients, cramped space, apart from other defects, is sufficient to make a new department necessary. His own view of the Insurance Act appears to be that it may diminish the number of general casualties, but he is inclined to think that the number of these regular out-patients which seek admission to the special departments may be much increased. As regards the latter it is satisfactory to learn that the ward adapted as a physical exercise department, with its mechanical aid apparatus, is justifying its cost, while the massage school is also progressing.

### THE NIGHT-BELL IN CANADA.

We recently discussed various substitutes for the old and in many ways unsatisfactory night-bell in private wards and urged the claims of electric lamps which are universal in America. In giving details of the equipment of the new Toronto General Hospital the *Hospital World* says: "The nurse-call equipment is very complete and embodies the most recent developments of the silent-call system. If a patient desires to call a nurse he simply presses a button upon which three Tungsten lamps showing the number of his bed flash at different places, at one of which the nurse is to be found." These lamps continue to burn until the call is responded to. It is the lack of details of this sort which impresses Canadian and American visitors with a sense of our conservatism, and now that the huge pile of the King's College Hospital is nearly finished it may be well to insist on their importance if the new hospital is to be in fact as well as in name a "show place" to Colonial and American officials on their arrival in this country.

### ST. KATHARINE'S HOSPITAL.

On more than one occasion in the past reference has been made in The Hospital to the case of St. Katharine's Hospital, Regent's Park. Public attention is likely to be called to the charity once more by the information which is now forthcoming that the Rev. John Hulbert Glover, formerly vicar of Kingsthorpe, Northants, and at his death a Brother of St. Katharine's Hospital, who died on February 9, at the age of ninety-one, left estate to the gross value of £167,655, of which £167,583 is net personalty.

### A DUKE'S GIFT OF AN ISOLATION HOSPITAL

A donation of £200 is announced from the Duke of Buccleuch towards the cost of a new infectious diseases hospital at Whitehill, near Dalkeith, for the use of the borough. The Duke has also presented, free, the ground for the hospital, extending to nearly two acres. The modern infectious diseases hospital attains such up-to-date efficiency that the new buildings for Dalkeith will be awaited with interest.

### NEW OFFICIALS AT WARRINGTON INFIRMARY.

The retirement of Mr. G. Dakin from the honorary treasurership of the Warrington Infirmary created a vacancy which has now been filled. Mr. Dakin held the post for twenty years, during which period he had the charge of the finances of the hospital, a task grown no less onerous as the years have passed. His successor, who was appointed at the recent annual meeting, is Mr. Roger C. Parr. An equally important change has been caused by the retirement of Mr. W. Peter Rylands from the chairmanship, which office he held for several years. The new chairman is Mr. James Henry Smethurst, who is a well-known worker for the hospital. The outlook for the new officials, though of course much complicated by the Insurance Act, is promising in the sense that though the maintenance expenses have necessarily increased since the extension of 1907, the income has been raised to an extent practically equal to the new charges, though an additional £600 a year is really needed if the institution is to pay its way. The financial result of the coming year will be eagerly awaited under the new management.

### THIS WEEK'S DRUG MARKET.

Business in the drug market has been practically at a standstill during the past week owing to the Easter holidays and few changes in value have taken place. Opium and cod-liver oil continue to have a declining tendency as regards prices. There is no alteration in the position of iodine and iodides, but the market is unsettled and a reduction in value is probable.

### TO OUR READERS.

Contributions are specially invited from any of our readers to these columns. They should deal with topical subjects and news. They must be authenticated for the information of the Editor only. Th minimum payment if published is 5s. There is no hard-and-fast rule as to space, but notes of about twenty lines in length are preferred.

